# Pharmacological insights into *Chukrasia velutina* bark: Experimental and computer‐aided approaches

**DOI:** 10.1002/ame2.12268

**Published:** 2022-09-01

**Authors:** Israt Jahan, Shahenur Alam Sakib, Najmul Alam, Mohuya Majumder, Sanjida Sharmin, A. S. M. Ali Reza

**Affiliations:** ^1^ Department of Pharmacy International Islamic University Chittagong Chittagong Bangladesh; ^2^ Department of Pharmacy East West University Dhaka Bangladesh

**Keywords:** anti‐nociceptive, antioxidant, antipyretic, *Chukrasia velutina*, cytotoxicity

## Abstract

**Background:**

*Chukrasia velutina* is an enthnomedicinally used plant reported to have significant medicinal values. The present study aimed to explore the pharmacological activities of bark methanol extract using in vitro, in vivo and in silico models.

**Methods:**

The study was designed to investigate the pharmacological effects of methanol extract of *Chukrasia velutina* bark (MECVB) through in vitro, in vivo and in silico assays. Analgesic activity was tested using formalin‐induced nociception and acetic acid‐induced writhing assays while the antipyretic effect was tested using yeast‐induced hyperthermia in mice model. The antioxidant effect was tested using the DPPH and reducing power assay and the cytotoxic screening was tested using the brine shrimp lethality bioassay. In addition, in silico studies were conducted using computer aided methods.

**Results:**

In the acetic acid‐induced writhing assay, the extract showed 28.36% and 56.16% inhibition of writhing for doses of 200 and 400 mg/kg, respectively. Moreover, a dose‐dependent formalin‐induced licking response was observed in both early and late phase. In yeast‐induced pyrexia, the MECVB exhibited (*p* < 0.05) antipyretic effect. The extract demonstrated an IC_50_ value of 78.86 μg/ml compared with ascorbic acid (IC_50_ 23.53 μg/ml) in the DPPH scavenging assay. The compounds sitosterol, 5,7‐dimethoxycoumarin and scopoletin were seen be effective in molecular docking scores against COX‐I (2OYE), COX‐II (6COX) and human peroxiredoxin 5 (1HD2). In ADME/T analysis, 5,7‐dimethoxycoumarin and scopoletin satisfied Lipinski's rule of five and thus are potential drug candidates.

**Conclusion:**

The bark of *Chukrasia velutina* showed significant analgesic and antipyretic properties and is a potential source of natural anti‐oxidative agents.

## Graphical abstract

Pharmacological activities of *Chukrasia velutina*.

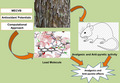

## 1 INTRODUCTION

Pain is a universal signal of many acute or chronic diseases like fever, inflammation, cancer and arthritis,^1^ presenting as an unpleasant sensation connected to cell damage and oxidative stress. It acts on the central or peripheral nervous system, mainly to promote the secretion of prostaglandin. Non‐steroidal anti‐inflammatory drugs (NSAIDs) are the most common type of drug for reducing the pain produced by chronic diseases. Antioxidant agents, mostly found in fruits, vegetables, nutritious food and so on, can alleviate oxidation. These agents exhibit phytochemical properties in various medicinal plants used in as herbal therapeutics. Free radicals are generated in many chronic diseases and are responsible for oxidation in cells and creating pain. They can be removed by antioxidant‐enriched food intake^2^; antioxidant compounds with phenolic properties, available in edible and non‐edible herbs, can scavenge the free radicals in the body thus alleviating pain.^3^

Pyrexia is a secondary symptom of inflammation, cardiovascular diseases, cell damage, malignancy, infection, and so on. When the body's general defense mechanism has created a pyrexic environment, pyrogens or infectious particles cannot survive. Damaged cells or foreign materials in the body initiate a rise in the production of pro‐inflammatory mediators. Subsequently, increases in the secretion of prostaglandin E2 near the hypothalamus region trigger the hypothalamus to raise the body temperature.^1^ NSAIDs are the most commonly prescribed drugs for pyrexia. They obstruct the release of COX‐2 materials and reduce the temperature of the body. Side effects can be reduced by using selective COX‐2 inhibitors, but the risk of adverse cardiovascular events needs serious consideration.^4^ Several medicinal plants have been used to inhibit the secretion of prostaglandins generated by COX‐2 and decrease the temperature of the body.

The phytochemicals that are obtained from these medicinal plants have quite powerful effects and fewer side effects than synthetic drugs.^5^ Traditional herbal medicines are the first line of medication for most of the people in underdeveloped countries because they are effective and more accessible than commercial synthetic medicines.^6^ Moreover, numerous drugs based on medicinal plants are used to treat various chronic diseases such as cancer, hypertension, diabetics, arthritis, inflammation and so on.^7^ In the twenty‐first century, within the socio‐economic context of different health services, the pharmaceutical markets encourage and support research into medicines from traditional medicinal plants.^8^

*Chukrasia velutina*, usually known as Chickrassy, belongs to the Meliaceae family^9^ and grows widely throughout south and eastern Asia. It exhibits a wide range of antimicrobial properties including antimalarial, antiprotozoal and other properties.^10^ The leaves and bark of the plant are also protective against astringent, diarrhea and pyrexia.^9^ Previous research has evaluated the antimicrobial efficacy of the essential oils found in the leaves^11^ which are also enriched with various potential phytochemicals with strong neuropharmacological activities.^12^ In addition, the bark powder has been shown to possess antimalarial and antibacterial properties.^9^ On the basis of previous investigations, we chose the bark of *C. velutina* for our current investigation of the plant's anti‐pyretic, analgesic, antioxidant and cytotoxic properties. Our investigations employed in vitro, in vivo and computational analyses such as molecular docking, pharmacokinetics properties and PASS prediction analysis. *C. velutina* contains potentially therapeutic compounds such as tubulin N, carapanolide Y, moluccensin Z1 and moluccensin Z2, velutinasin J and chukvelutilide A1,^10^ and many secondary metabolites such as terpenes, triterpenes, limonoids, coumarins,^9^ sitosterol, 5,7‐dimethoxycoumarin, scopoletin, melianone, tannic acid, tabulalin and tabulalide A.^13^

## 2 MATERIALS AND METHODS

### 2.1 Chemicals and reagents

The following chemicals and drugs were employed in these studies: Folin‐Ciocalteu (FCR), gallic acid, ascorbic acid, ferrous chloride, sodium carbonate (saline), potassium ferricyanide, trichloroacetic acid, methanol, DMSO, sodium chloride, Paracetamol, yeast, chloramphenicol, tween‐80, formaldehyde, acetic acid, diclofenac sodium.

### 2.2 Plant collection and extraction

The bark of *C. velutina* was acquired from the forest area of Bangladesh Forest Research Institute, Chittagong, Bangladesh, during the month of April, 2019. Its identity was confirmed by an expert at the Bangladesh Forest Research Institute (BFRI). An extract sample (accession no. BFRI‐109) has been preserved in the herbarium of the BFRI. The bark samples were air dried at room temperature and ground to coarse powder using an electric grinder. The resulting coarse powder (500 g) was soaked in methanol at a 1:3 volume ratio. After 10 days, the mixture was strained through Filter Paper number 1. The methanol solvent was then vaporized in a water bath at 60–65°C temperature, resulting in 18.27 g of crude extract (yield w/w, 4.08%) termed methanol extract of *C. velutina* bark (MECVB). The extract was poured in a glass vial and stored in a refrigerator at 4°C for use in the anti‐nociceptive, antipyretic, antioxidant and cytotoxic effect studies.

### 2.3 Experimental animals

Swiss albino mice were purchased from Jahangir Nagar University, Savar, Dhaka, Bangladesh. The mice were all male and weighed about 25–30 g. The animals were placed in polypropylene cages in controlled laboratory conditions with a room temperature of 25 ± 2°C, a relative humidity of 55–60%, a 12 h light/dark cycle and standard laboratory food supplied ad libitum. All tests using the mice were conducted in a peaceful environment and the animals were adapted to the laboratory conditions prior to the 10 days of the assays, in accordance with internationally accepted guidelines for use of laboratory animals, namely the National Institutes of Health (NIH) and International Council for Laboratory Animal Science (ICLAS) guidelines. The procedures used in the present studies were reviewed and accepted by the P&D Committee of the Department of Pharmacy, International Islamic University Chittagong, Bangladesh (reference number: Pharm‐P&D‐61/08′16‐125).

### 2.4 Acute toxicity test

Acute toxicity analysis was conducted under standard laboratory conditions following the Organization for Environmental Control Development guidelines.^12^ All doses of MECVB (5, 10, 50, 100, 200, 400, 800, 1000 mg/kg) were administered orally to the experimented animals (*n* = 6). The negative control group was administered 1% Tween 80 solution. All experimental animals were closely observed for the next 24 h and mortality, behavioral changes, and toxicity were recorded.

### 2.5 Dosing groups for in vivo studies

In this study, mice were distributed randomly into four groups of six mice (*n* = 6). The control group received 1% Tween‐80 in distilled water and the standard NSAID drug diclofenac Na (10 mg/kg body weight) and paracetamol (100 mg/kg body weight) were received by the positive control group (Paracetamol group). The last two groups were given MECVB at doses of 200 and 400 mg/kg body weight previously ascertained through acute toxicity analysis.

### 2.6 Anti‐nociceptive analysis

#### 2.6.1 Acetic acid‐induced writhing assay

The acetic acid‐induced writhing assay was used to evaluate the peripheral analgesic effect of MECVB. The mice were treated with diclofenac Na (10 mg/kg, body weight) or MECVB (200 and 400 mg/kg, body weight). Thirty minutes after administration 0.6% acetic acid (10 mg/kg, body weight) was introduced intraperitoneally into the mice. After 5 min, the writhing was counting for 15 min and the number of writhes—contraction of the abdomen, elongation of the body, twisting of the trunk and extension of the pelvis was compared to the control group writhing.^4^ The anti‐nociceptive effect was presented as the percentage inhibition of writhing calculated using the following equation:

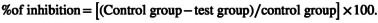




#### 2.6.2 Formalin‐induced nociception

The formalin‐induced licking test was performed on mice following injection of 20 μl 2.5% formalin solution into the subcutaneous region of the right hind paw 30 min after administration of 200 and 400 mg/kg body weight MECVB. The pain sensation was considered to reflect of the nociceptive sensation, and the overall time spent in the behavioral responses to nociception when the mice were licking or biting the injected paw was recorded for total of 30 min, with the initial 5 min (0–5) referred to as the neurogenic phase and the last 15 min (15–30) referred to as the late or inflammatory phase.^4^ The percentage inhibition was calculated using the equation described in the previous section.

### 2.7 Antipyretic effect

#### 2.7.1 Yeast‐induced hyperthermia in mice

The anti‐pyretic effect of the extract was assessed using fever induced by Brewer's yeast following the established method in mice with some changes. The experimental animals weighed 25–30 g and were acclimatized to laboratory conditions and given no food 12 h before the test. The rectal temperature of the mice was recorded with a digital thermometer and pyrexia was produced in all mice by injecting a 20% aqueous suspension of Brewer's yeast (10 mg/kg). All the mice groups were fasted overnight but given free access to drinking water. After 24 h, the rectal temperature of the mice was recorded. During this time, pyrexia was induced in the mice with a mean temperature increase of more than 0.5°F; animals that exhibited an increase in temperature of less than 0.5°F were excluded from the experiment. All the animals in the experimental groups were then administered MECVB extract at doses of 400 and 200 mg/kg, body weight. After drug and extract administration, the rectal temperature of the mice was recorded at 0, 1, 2, and 3 h.^14^

### 2.8 Antioxidant effect

#### 2.8.1 DPPH free radical scavenging assay

The free radical scavenging effect of MECVB was investigated using DPPH. A 0.1 ml sample of DPPH in a 1 mM methanol solution of was prepared and mixed with various concentrations of MECVB (0.0625 to 1 mg/ml). The solutions were incubated at room temperature for about 30 min. After incubation, the absorbance of the resulted solution was measured at 517 nm. Ascorbic acid was used as a reference material.^15^ The scavenging effect of DPPH free radical was calculated using the following equation:

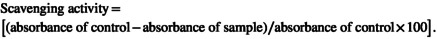




#### 2.8.2 Total phenolic content

The total phenol content (TPC) in the MECVB was established using the FCR method. TPC was assessed spectrophotometrically by measuring absorbance at 760 nm.^16^ A 1 ml methanol solution of extract was mixed with 2.5 ml FCR and 2.5 ml Na_2_CO_3_ and incubated at 25°C for about 20 min, after which the absorbance of the solution was measured. Three measurements were taken to determine the mean absorbance, providing the TPC value of extract solution. A blank solution was prepared with methanol instead of extract solution. Using gallic acid as a standard, the same procedure was followed and the calibration line was set up again. TPC was presented in terms of gallic acid equivalents (mg GaA/g per extract).

#### 2.8.3 Reducing power analysis

The reducing power of MECVB was determined using the Qyaizu method. Following this his method, 1 ml methanolic solution of the extract was added to 2.5 ml phosphate buffer and 2.5 ml 1% potassium ferricyanide. This mixture was then incubated for 20 min, at about 50°C. After incubation, 2.5 ml of 10% TCA was added and centrifuged. Then 0.5 ml FeCl_3_ and 2.5 ml distilled water were added to the supernatant and the absorbance was measured at about 700 nm. The absorbance of the sample increased when the concentration of extract increased.^16^

### 2.9 Cytotoxic screening

Cytotoxic screening of MECVB was performed using the brine shrimp lethality bioassay. After hatching brine shrimp, saline was prepared from distilled water and sea salt (adding a small amount of DMSO). MECVB was serially diluted to concentrations between 320 and 10 μg/ml, using 5 mg extract in DMSO. Five milliliters of sea salt were added to all the test tubes. Finally the same amount of brine shrimp was added to all test tubes. After 24 h, the number of brine shrimp alive was checked and the mortality rate of brine shrimp recorded.^17^

### 2.10 In silico studies

#### 2.10.1 Selected compounds for molecular docking

Seven phytochemicals present in the bark of *C. velutina* were picked from a literature review^13^: sitosterol (PubChem CID: 222284), 5,7‐dimethoxycoumarin (PubChem CID: 2775), scopoltin (PubChem CID: 5280460), melianone (PubChem CID: 99981), tannic acid (PubChem CID: 16129778), tabulalin (PubChem CID: 101355658), tabulalide A (PubChem CID: 101355662).

#### 2.10.2 Protein and ligand preparation

The 3D (three‐dimensional) and 2D (two‐dimensional) crystal structures were installed from the Protein Data Bank (https://www.rcsb.org)^18^ for the following proteins: cyclooxygenase enzyme‐1 (pdb: 2OYE),^19^ cyclooxygenase enzyme‐2 (pdb: 6COX)^19^ and human peroxiredoxin 5 (pdb: 1HD2).^20^ These proteins were prepared as previously described using the wizard of Schrödinger‐Maestro v10.1 (Schrödinger, LLC New York, NY, USA). Alternatively, the chemical structure of the seven phytocompounds of *C. velutina* were retrieved from the PubChem database (https://pubchem.ncbi.nlm.nih.gov). The ligands to be docked were then made with the Lig‐Prep tool ingrained in Maestro 2015, followed by neutralization at pH 7.0 ± 2.0 using Epik and understated by force field OPLS‐2005.

#### 2.10.3 Receptor grid generation

Receptor grids were calculated for prepared proteins so that various ligand poses bind within the predicted active site during docking. In Glide, grids were generated in a way to keep the default parameters of van der Waals scaling factor 1.00 and charge cutoff value 0.25 subjected to OPLS 2005 force field. A cubic box of specific dimensions centered on the centroid of the active site residues was generated for receptor. The bounding box was set to 14 Å × 14 Å × 14 Å for docking experiments.

#### 2.10.4 Glide standard precision ligand docking

Using Glide of Schrödinger‐Maestro v 10.1, standard precision, flexible ligand docking with receptor was carried out with sanctions applied for any non‐cis/trans amide bonds. The scaling factor for Van der Waals and partial charge cutoff for ligand atoms were chosen to be 0.80 and 0.15, respectively. Final scoring was performed on energy‐minimized poses and displayed as a Glide score. The best‐docked pose with the lowest Glide score value was recorded for each ligand.

#### 2.10.5 Investigation of ADME/T properties

The pharmacokinetics properties of these phytochemicals was analyzed by SwissADME (http://www.swissadme.ch) where the compounds were assessed for drug‐like parameters using Lipinski's rule of five. If these constituents supported the rule with molecular weight ≤ 500, H‐bond acceptors ≤10, H‐bond donor ≤5, lipophilicity ˂ 5, and molecular refractivity from 40 to 130, the compounds were considered a good drug candidate.

#### 2.10.6 PASS prediction

The major compounds were assessed using the computer aided tool PASS online (http://www.way2drug.com) to identify possible biological properties. The potential biological properties of these compounds were expressed as *P*_*a*_ (probable activity) and *P*_*i*_ (probable inactivity) where the values for *P*_*a*_ and *P*_*i*_ vary from 0.000 to 1.000 and *P*_*a*_ ˃ *P*_*i*_ indicated the possible biological activities of the selected phytochemicals.

### 2.11 Statistical analysis

The results obtained were expressed as the mean ± SEM (standard error of the mean) of six (*n* = 6) animals. Statistical analysis was performed utilizing GraphPad Prism version 6.01. For statistical analysis, one‐way analysis of variance ANOVA followed by post‐hoc Dunnett's (*t* test) was used to compare the test samples with the control. *p* < 0.05 and *p* < 0.01 were considered statistically significant when the data were compared with the negative control (1% Tween 80 solutions) group in the in vivo study.

## 3 RESULTS

### 3.1 Acetic acid‐induced writhing test

**FIGURE 1:**
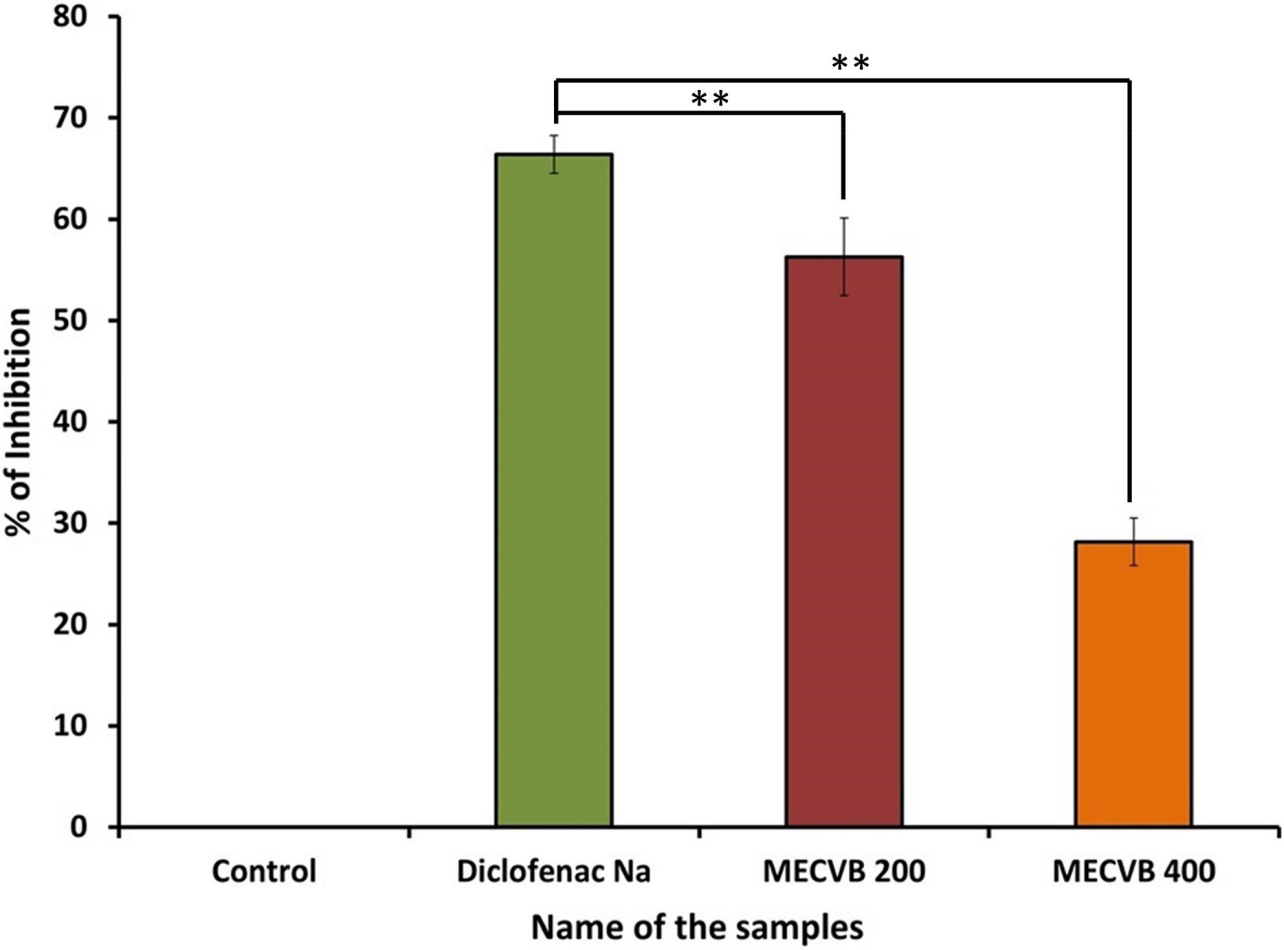
Effect of MECVB in acetic‐acid induced writhing test. Values are mean ± S.E.M. **p<0.01, significantly different from Diclofenal Na; ANOVA followed Dunnett’s test (*n* = 6, per group). Where, MECVB = Methanol extract of *C. velutina* bark.

In the acetic acid‐induced writhing test, the numbers of writhes were reduced in animals treated with MECVB at doses of 200 and 400 mg/kg. The effects of these two doses of extract and of diclofenac Na were compared with controls to determination of their significance level. The values for percentage inhibition of writhing induced by the extract (200, 400 mg/kg) and diclofenac Na (10 mg/kg) were 28.36%, 56.16% and 66.66% respectively, and the results were statistically significant (*p* < 0.01) as shown in (Figure 1).

### 3.2 Formalin‐induced nociception

**FIGURE 2:**
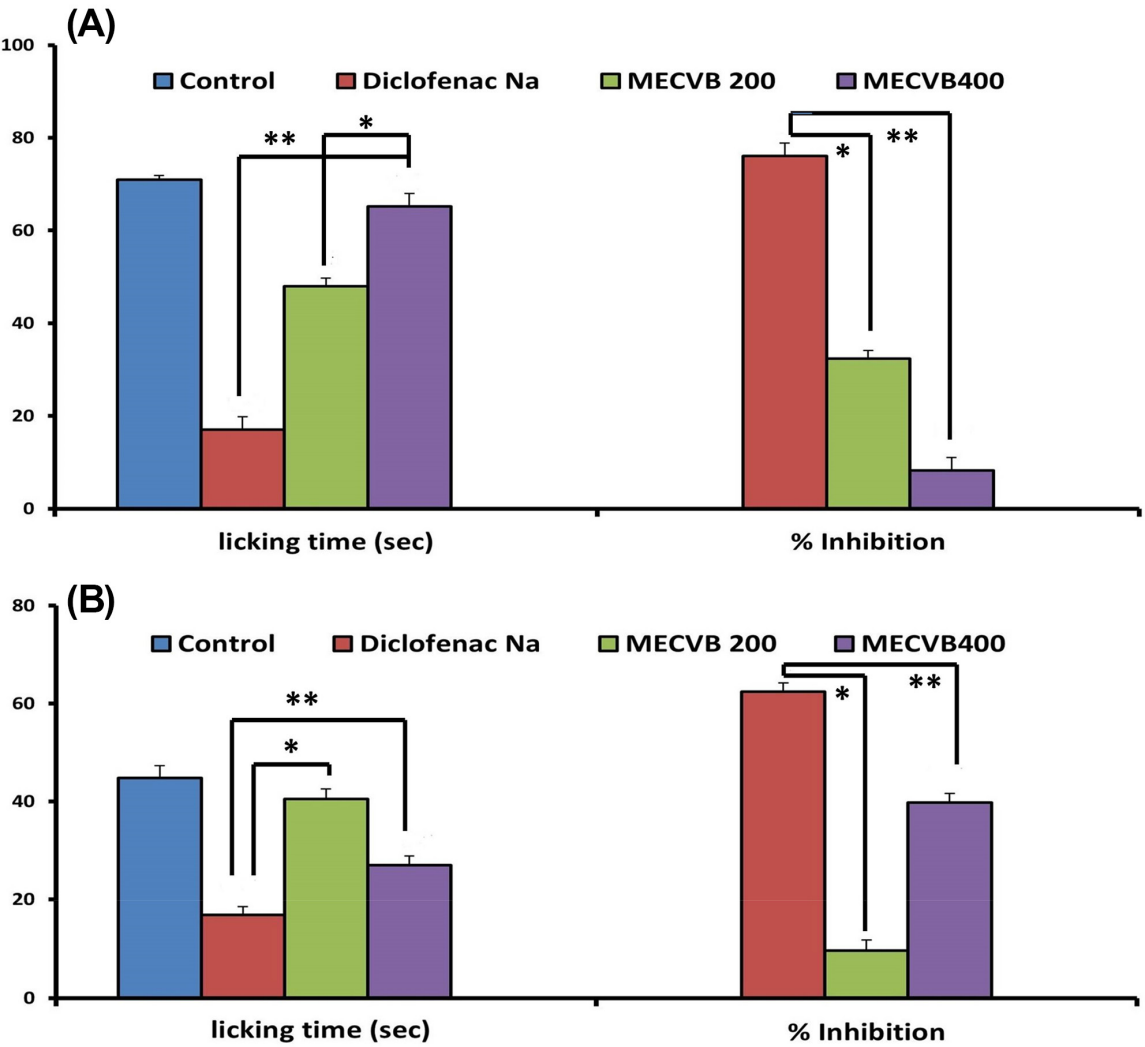
Effect of MECVB on formalin test (A) first phase and (B) second phase. Values are mean ± S.E.M. *p<0.05 and **p<0.01, significantly different from Diclofenal Na; ANOVA followed Dunnett’s test (*n* = 6, per group). MECVB = Methanol extract of *C. velutina* bark.

In the assessment of formalin‐induced nociception, the effect of orally administered MECVB extract was dose dependent. Treatment with MECVB at doses of 400 mg/kg and 200 mg/kg significantly decreased the licking of mice in the early neurogenic phase by 32.39% (*p* < 0.01) and 8.17% (*p* < 0.01), respectively, and in the late inflammatory phase by 39.54% (*p* < 0.01) and 9.54% (*p* < 0.01), respectively, when compared with diclofenac Na. Diclofenac Na, which used as a standard drug as a dose of 10 mg/kg body weight, significantly decreased the licking of mice in both the inflammatory and neurogenic phases by 75.80% and 62.16% (*p* < 0.05) respectively (Figure 2).

### 3.3 Antipyretic effect

Hyperthermia was produced in the mice by subcutaneous administration of Brewer's yeast. The rectal temperatures of the mice increased 2 h later (Table 1). Methanol extract at doses of 200 and 400 mg/kg and the standard drug paracetamol were then administered. The resulting antipyretic effects are shown in (Table 1). Temperatures were recorded from 1 to 3 h after drug administration and both the MECVB extract and paracetamol treatment groups showed a significant effect (*p* < 0.05, *p* < 0.01), with a reduction in rectal temperature and decreased hyperthermia. The antipyretic effects of MECVB extract at 400 mg/kg and the standard drug paracetamol differed significantly compared with the positive control.

**TABLE 1:** Antipyretic effects of MECVB in yeast‐induced pyrexia

Treatment	Doses (mg/kg)	Initial rectal temperature of mice (°F)	Rectal temperature of mice after 24 h (°F)			
			0 h	1 h	2 h	3 h
Control	–	98.0	100.6 ± 0.11	100.7 ± 0.06	100.7 ± 0.05	100.6 ± 0.10
Paracetamol	100	97.8	100.5 ± 0.12	99.83 ± 0.23^a^	99.14 ± 0.05	98.76 ± 0.11
MECVB	400	96.9	99.59 ± 0.28^a^	98.72 ± 0.17	97.53 ± 0.21	96.74 ± 0.14
MECVB	200	96.3	100.2 ± 0.06	99.47 ± 0.12^b^	98.28 ± 0.15	97.76 ± 0.08

*Note*: Values are the mean ± SEM (*n* = 6). Data were analyzed by one‐way analysis of variance (ANOVA), Values with *p* < 0.05 were considered as significant. ^a,b^Superscript letters indicate that values are significantly different from each other.

Abbreviation: MECVB, methanol extract of *C. velutina* bark.

### 3.4 DPPH free radical scavenging effect

In the analysis of the DPPH radical scavenging effect of MECVB, the extract showed antioxidant capacity (Table 2). In this test, the capacity to decolorize DPPH by freeing the radical depended on the concentration of extract. The IC_50_ value of ascorbic acid was 23.53 μg/ml and the IC_50_ of MECVB was 78.86 μg/ml.

**TABLE 2:** DPPH scavenging activity of ascorbic acid and MECVB

Sample	IC_50_ (μg/ml)
Ascorbic acid (standard)	23.53
MECVB	78.86

Abbreviation: MECVB, methanol extract of *C. velutina* bark.

### 3.5 Total phenolic content

Phenol is a potent plant compound with significant antioxidant activity, containing hydroxyl groups that act as free radical terminators. The total phenol content of MECVB was expressed as gallic acid equivalent (GAE) mg/g dry extract and was 588.569 ± 0.113 GAE mg/g dry extract (Table 3).

**TABLE 3:** Determination of total phenolic content (TPC) of MECVB as GAE mg/gm of dry extract

Sample solution of MECVB (μg/ml)	Weight of dry extract per ml, (mg)	Absorbance at 760 nm	GAE conc. C (μg/ml)^b^	GAE conc. C (mg/ml)	TPC expressed as GAE, (*A* = [*c* × *v*]/*m*) (mg/g)	Mean ± SEM
500	0.001	2.937	294.18	294 186.27	588.37	588.56 ± 0.113
500	0.001	2.938	294.28	294 284.31	588.56	
500	0.001	2.939	294.38	294 382.35	588.76	

Abbreviation: MECVB, methanol extract of *C. velutina* bark.

### 3.6 Reducing power capacity

**FIGURE 3:**
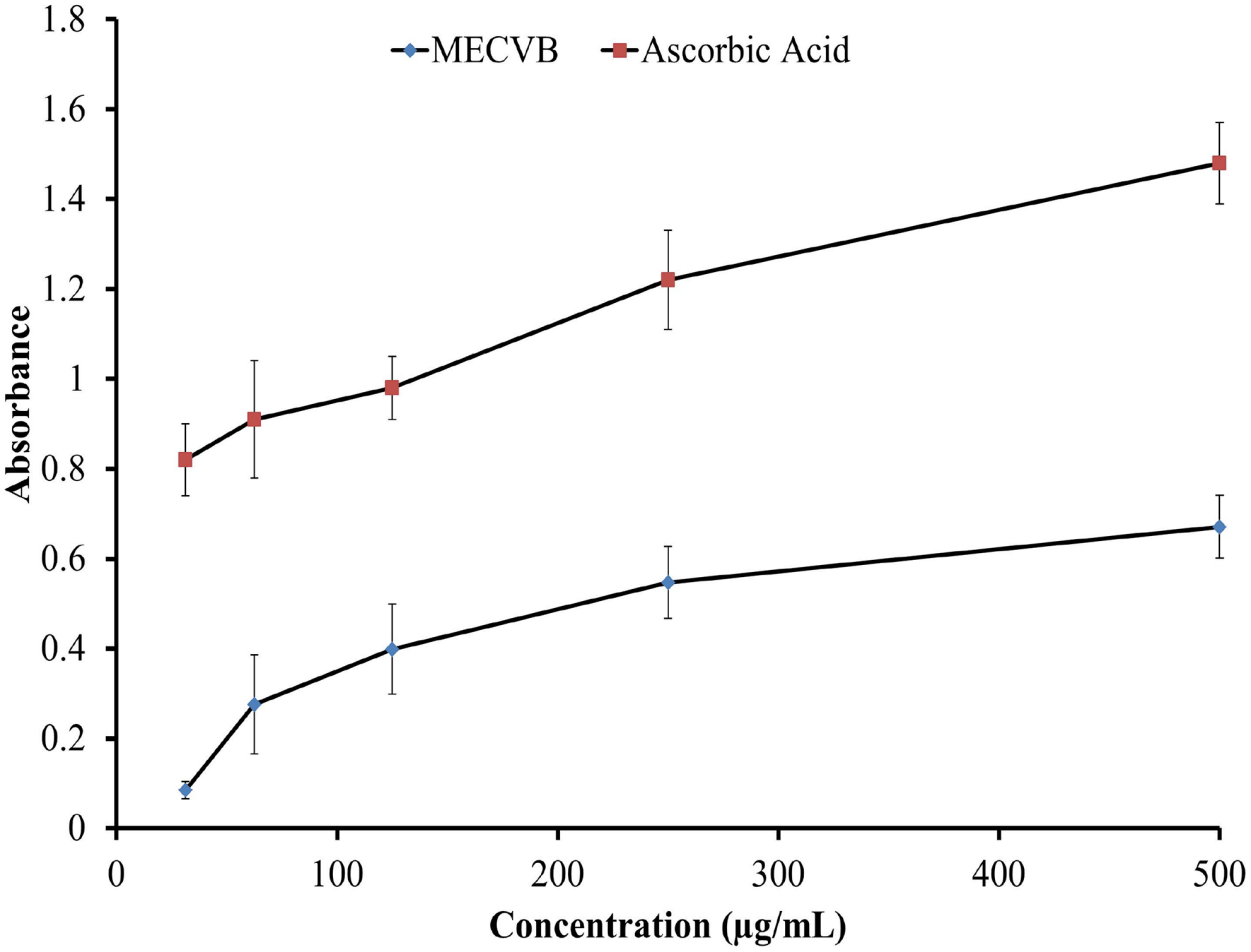
Reducing power capacity of ascorbic acid and MECVB.

The antioxidant effect of a compound is connected with its reducing power. Compounds displaying reducing power decrease oxidation by donating a hydrogen atom. The reducing power is also related to the presence of reductants in antioxidants that reduce the Fe^3+^ ferricyanide complex to the Fe^2+^ ferrous form. The amount of ferrous form is determined spectrophotometrically by taking the absorbance at 700 nm. The increase in the MECVB extract absorbance correlated with the concentration of sample solution, showing that the plant had significant reducing power. The MECVB extract at concentrations of 500, 250 and 125 μg/ml gave absorbance values of 0.671, 0.547 and 0.299, respectively, compared with the reference standard ascorbic acid absorbance values of 1.48, 1.22 and 0.98 at similar concentrations (Figure 3).

### 3.7 Cytotoxic screening

The cytotoxic effect of MECVB was estimated using the freshly hatched brine shrimp lethality bioassay. The activity of the extract was dose dependent. In this assay, 10 μg/ml of extract exhibited the lowest mortality (20%) and 320 μg/ml demonstrated the highest (70%) compared with chloramphenicol used as standard. At the same concentrations chloramphenicol produced 10% and 100% mortality, respectively. The LC_50_ value of the extract was calculated to be 139.95 μg/ml (Table 4), which proved that there is a potent bioactive compound present in MECVB extract.

**TABLE 4:** In vitro cytotoxicity activity of MECVB using the brine shrimp lethality assay

Sample	Conc. of extract (μg/ml)	Number of shrimps taken	Number of live shrimps	Number of dead shrimps	% Mortality	LC_50_ (μg/ml)
MECVB	320	10	03	07	70	139.95
	160	10	04	06	60	
	80	10	05	05	50	
	40	10	06	04	40	
	20	10	07	03	30	
	10	10	08	02	20	
Chloramphenicol	320	10	00	10	100	72.33
	160	10	01	09	90	
	80	10	03	07	70	
	40	10	05	05	50	
	20	10	07	03	30	
	10	10	09	01	10	

### 3.8 Molecular docking

**FIGURE 4:**
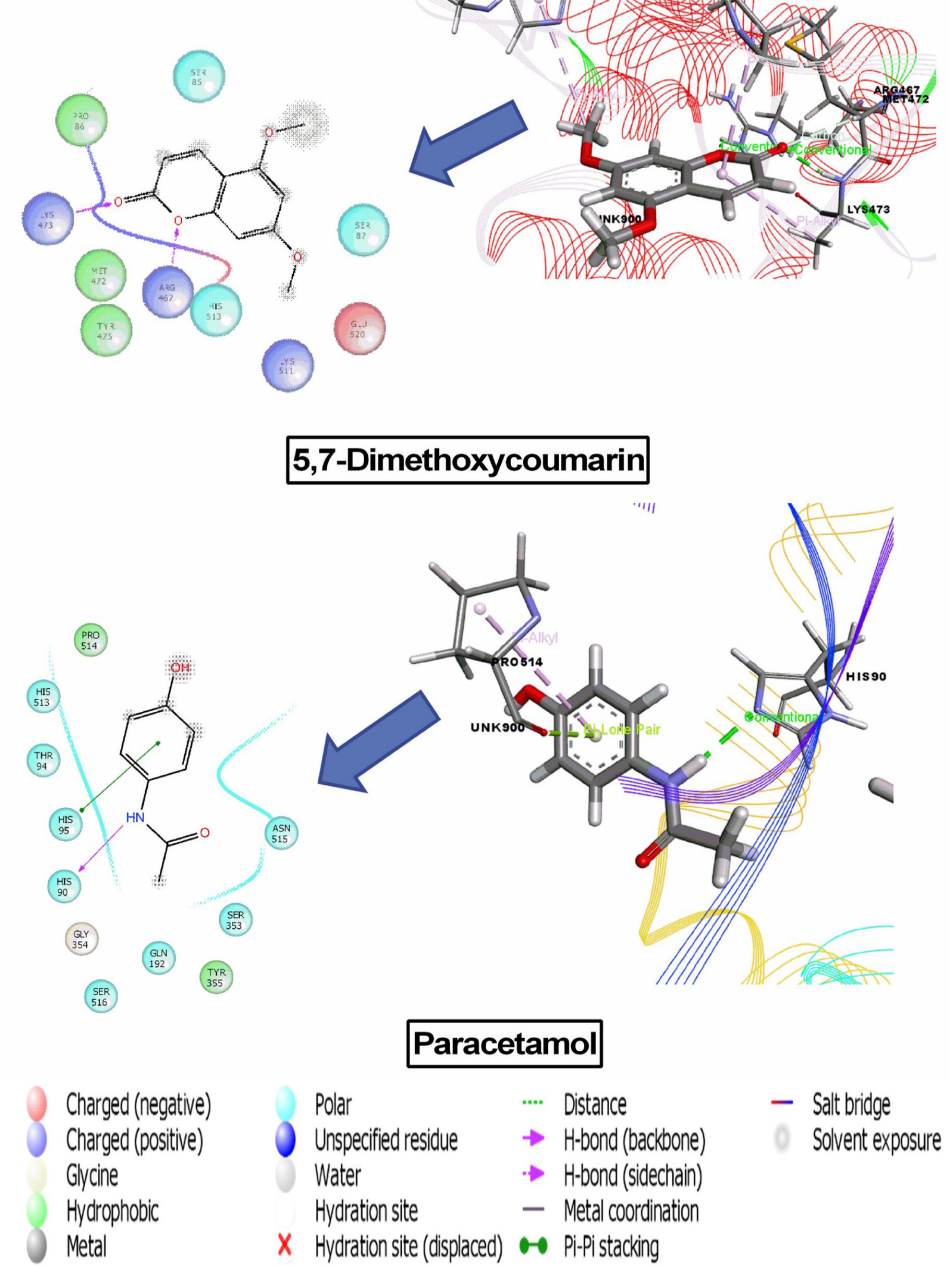
Docking results of 5,7‐dimethoxycoumarin and paracetamol with COX‐1 ((PDB ID: 2OYE) for analgesic activity.

**FIGURE 5:**
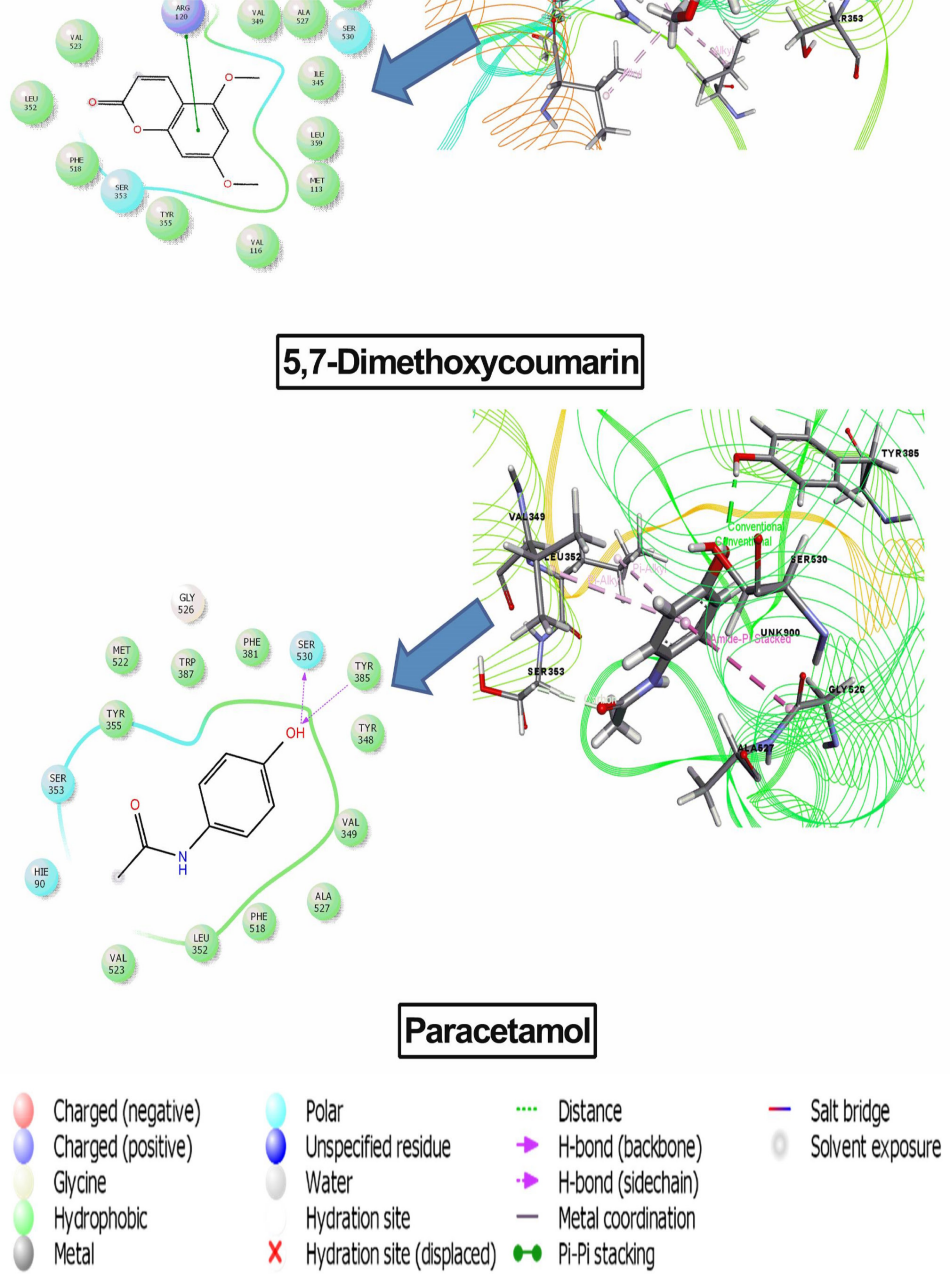
Docking results of 5,7‐dimethoxycoumarin and paracetamol with COX‐2 (PDB ID: 6COX) for analgesic activity.

**FIGURE 6:**
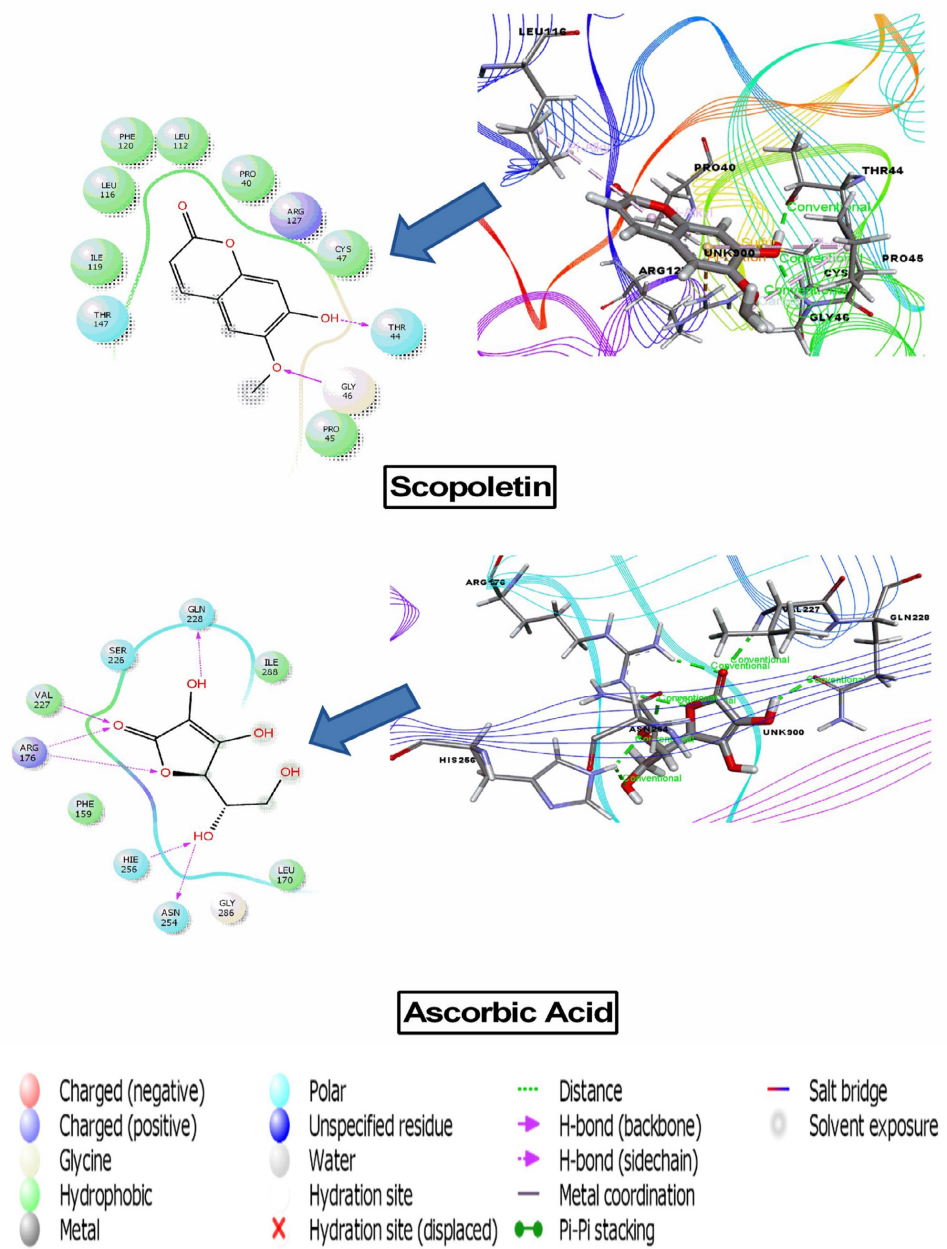
Docking results of scopoletin and ascorbic acid with 1HD2 receptor for antioxidant activity.

The result of docking analysis is presented in (Table 5) and the best docking score interaction figures are shown in (Figures 4, 5, 6). In the case of the analgesic and antipyretic studies, three compounds docked with cyclooxygenase enzyme‐1 (PDB ID: 2OYE; docking score range −4.26 to −1.36 kcal/mol) and two compounds docked with cyclooxygenase enzyme‐2 (PDB ID: 6COX; docking score range −6.57 to −6.30 kcal/mol). Among the seven compounds tested, three showed antioxidant activity with a docking score range of −4.90 to −1.40 kcal/mol. The results show that 5,7‐dimethoxycoumarin displayed the highest docking scores: −4.29 and − 6.57 kcal/mol against COX‐1 and COX‐2, respectively. Scopoletin exhibited the best score, −4.90 kcal/mol, against human peroxiredoxin 5. However, melianone, tannic acid, tabulalin and tabulalide A did not interact with the protein or enzyme or receptor.

**TABLE 5:** Docking score of some selected compounds isolated from MECVB. Cyclooxygenase enzyme‐1 (PDB ID: 2OYE), cyclooxygenase enzyme‐2 (PDB ID: 6COX) and human peroxiredoxin 5 (PDB ID: 1HD2) were used to test analgesic, antipyretic and antioxidant activity, respectively

Compound name	Docking scores		
	COX‐1 (2OYE)	COX‐2 (6COX)	Antioxidant (1HD2)
Sitosterol	−1.36	–	−1.40
5,7‐Dimethoxycoumarin	**−4.29**	**−6.57**	−4.71
Scopoletin	−4.26	−6.30	**−4.90**
Melianone	–	–	–
Tannic acid	–	–	–
Tabulalin	–	–	–
Tabulalide A	–	–	–
Standard drugs (paracetamol/ascorbic acid)	−4.843	−4.656	−5.134

*Note*: Docking scores in kcal/mol; bold highlighted text indicates the highest score.

### 3.9 ADME/T analysis

In Table 5, two compounds (5,7‐dimethoxycoumarin and scopoletin) present in the bark of *C. velutina* exhibited a promising docking score against the receptors selected for antipyretic, anti‐nociceptive and antioxidant activity. In Table 6, these two phytochemicals also followed Lipinski's rule of five indicating that both 5,7‐dimethoxycoumarin and scopoletin have potential pharmacokinetics properties such as absorption, distribution, metabolism and excretion.

**TABLE 6:** Analysis of the pharmacokinetics properties of scopoletin and 5,7‐dimethoxycoumarin

Compound	Molecular weight (g/mol)	H‐bond acceptor	H‐bond donor	Log P	Molecular refractivity	Rule of five violation
5,7‐Dimethoxycoumarin PubChem CID: 2775	206.19	4	0	1.92	55.47	0
Scopoletin PubChem CID: 5280460	192.17	4	1	1.52	51.00	0

### 3.10 PASS prediction

The compounds 5,7‐dimethoxycoumarin and scopoletin were analyzed with the PASS online tool. These compounds showed promising activity (*P*_*a*_ ˃ *P*_*i*_) for pharmacological properties such as antipyretic, analgesic and antioxidant activities (Table 7). For scopoletin, the *P*_*a*_ value was higher than *P*_*i*_ for anti‐nociceptive (0.425), antipyretic (0.575) and antioxidant (0.540) effects; and for 5,7‐dimethoxycoumarin, the *P*_*a*_ values for the antipyretic (0.463), anti‐nociceptive (0.357) and antioxidant (0.442) effects were higher than the *P*_*i*_ values.

**TABLE 7:** Pass prediction of biological activities of 5,7‐dimethoxycoumarin and scopoletin

Compound	Biological activity	*P*_*a*_	*P*_*i*_
5,7‐Dimethoxycoumarin	Oxidoreductase inhibitor	0.734	0.012
	Neurotransmitter antagonist	0.654	0.005
	Free radical scavenger	0.586	0.006
	Anti‐inflammatory	0.587	0.035
	Antipyretic	0.463	0.018
	Anti‐nociceptive	0.357	0.141
	Antioxidant	0.442	0.009
Scopoletin	Oxidoreductase inhibitor	0.790	0.007
	Neurotransmitter antagonist	0.692	0.004
	Free radical scavenger	0.742	0.003
	Anti‐inflammatory	0.629	0.026
	Anti‐nociceptive	0.425	0.090
	Antipyretic	0.575	0.008
	Antioxidant	0.540	0.005

## 4 DISCUSSION

A lot of medicinal plants are used in traditional medicine all over the world to reduce pain and treat hyperthermia; they can also play a vital role in counteracting oxidation and cytotoxicity in cells.^21^ The present study was designed to investigate the anti‐nociceptive, antipyretic, antioxidant and cytotoxic effects of a methanol extract of *C. velutina* bark on pain stimulation. Pain is currently a major global health issue evident as headaches, cancer, inflammation, arthritis, osteoporosis and fever. Many people are affected by acute or severe pain produced by release of COX‐1, COX‐2, prostaglandin, lipoxygenase, serotonin, histamine etc.^1^ In our study the anti‐nociceptive effect of MECVB was estimated in two mice models providing a response to noxious stimuli resulting from chemically induced tissue damage.^4^ The assignment of analgesic drugs depended on their mechanism of action on the peripheral or central nervous systems. The pain sensation in the acetic acid‐induced writhing response was demonstrated by triggering a localized inflammatory response resulting from the release of free arachidonic acid from tissue phospholipids via cyclooxygenase (COX‐1 and COX‐2) and prostaglandins, specifically PGE2 and PGF2 biosynthesis; the level of lipoxygenase products may also increase in peritoneal fluids.^22^ Intraperitoneal administration of MECVB significantly reduced the writhing induced by acetic acid. Our findings substantiate a strong peripheral analgesic activity of MECVB. The mechanism of action may be mediated by inhibition of cyclooxygenase activity or prostaglandin synthesis.

The formalin‐induced nociception model provides a long‐lasting pain with peripheral inflammation and central sensitization.^4^ This protocol involves a biphasic reaction consisting of an early (neurogenic) phase and a late (inflammatory) phase. It creates mostly neurogenic inflammation followed by sharing of kinins and leukocytes, including their pro‐inflammatory factors with prostaglandins.^1^ It is hypothesized that acute inflammation results from the consequences of formalin‐induced cell damage, which distributes endogenous mediator production.^23^ This study showed that the oral administration of bark extract produced an analgesic effect against both the neurogenic and inflammatory phases of formalin‐induced nociception. The intensity of pain in the experimental paradigm was evaluated by observing specific behavioral responses such as licking and the observations were converted to numerical values.^24^ Administration of MECVB at different doses significantly inhibited the pain response in both phases as confirmed by reduced licking behavior, but the effect was more prominent in the second phase. Reduced licking time in both phases therefore indicates a possible interaction with neurogenic and inflammatory pain modulators.

Brewer's yeast is an exogenous pyrogen which produces pathogenic fever by binding to lipopolysaccharide binding protein and results in the release of different cytokines like interleukin‐1, −6, tumor necrosis factor alpha and prostaglandins. These pro‐inflammatory mediators cross the blood–brain barrier and act on the hypothalamus causing the release of prostaglandin E2, which is produced through the action of cyclo‐oxygenase‐2 and thus increases the body temperature.^25^ In this study, a potential antipyretic effect was observed for the 200 and 400 mg/kg doses of MECVB and the effect was similar to that of paracetamol.

Oxidation is responsible for many diseases related to pain such as cancer, infection, arthritis, cardiovascular diseases and type‐2 diabetes.^16^ Antioxidant containing chemical compounds have been shown to reduce oxidation in cells of the body.^26^ In the present study, we evaluated the antioxidant effect of MECVB. The antioxidant potency of the extract was demonstrated using the DPPH scavenging free radical assay, total phenol content and the reducing power assay.

Many disorders such as inflammation, pain and neurodegenerative diseases are related to free radical activity.^27^ Antioxidants have the ability to scavenge these free radicals and are therefore useful for management of these disorders.^28^ The DPPH scavenging free radical assay is useful to determine the antioxidant effect of plant extracts.^16^ In this assay, DPPH, a stable free radical, was reduced by MECVB extract at a significant rate compared with the ascorbic acid standard, which has good antioxidant properties, decreasing free radicals by donating hydrogen atoms and converting the DPPH into a yellow colored α,α‐diphenyl‐β‐picryl hydrazine.^29^ The radical scavenging potency of antioxidants is quantified as the extent of discoloration of DPPH.^16^ MECVB extract has the capacity to neutralize DPPH by donating electrons or hydrogen atoms and converting DPPH to α,α‐diphenyl‐β‐picryl hydrazine.^16^ The scavenging potential of phenolic compound can be measured as the amount of scavenging of free radicals by their phenolic hydroxyl groups. The methanol extract of *C. velutina* bark is a good source of phenolic compounds which could break the chain reaction in oxidation and reduce the incidence of a lot of diseases. The reducing power of phytochemical compounds mainly depends on their concentration; when the concentration is increased, the absorbance is also raised.^30^ The antioxidant effect of *C. velutina* bark was demonstrated via analysis of the reducing power through reduction of the ferricyanide complex to the ferrous form and was greater than that seen in a previous study.^31^

The cytotoxic effect of *C. velutina* was evaluated at different concentrations compared with chloramphenicol. A brine shrimp lethality bioassay was conducted to test cytotoxicity,^32^ and measured mainly the mortality rate of brine shrimp at different concentration.^33^ Freshly hatched nauplli were be used in this assay and the MECVB extract was shown to be lethal. This effect should be further investigated in cancer cells.

In computational analysis, molecular docking investigations have been widely utilized to predict the biological activities of compounds isolated from plants. This analysis provides not only the possible mechanism of action but also the docking modes inside the binding pocket of the protein or enzyme or receptor.^34^ In ongoing investigation, two cyclooxygenase‐1 (COX‐1) and cyclooxygenase‐2 (COX‐2) receptors have been shown to be responsible for nociception and pyrexia. Moreover, these COX‐1 and COX‐2 receptors have various mechanical pathways in the human body which are implicated in several chronic diseases. In our analysis, the 2OYE and 6COX enzymes were utilized for COX‐1 and COX‐2, respectively, and the 1HD2 enzyme responsible for oxidation was used. Based on the results described above, the phytochemicals sitosterol, 5,7‐dimethoxycoumarin and scopoletin may be responsible for analgesic, antipyretic and antioxidant activities on the basis of their interactions with targeted proteins, enzymes or receptors. From a literature review, we found that sitosterol has anti‐inflammatory, antipyretic,^35^ anti‐diabetic, antioxidant^36^ and cytotoxic effects.^37^ Another phytocompound, 5,7‐dimethoxycoumarin has been reported to have miscellaneous pharmacological activities including analgesic,^38^ antibacterial, anticoagulant,^39^ anti‐diabetic activities and has been used in treatment of Alzheimer's disease.^40^ In addition, scopoletin possesses a wide range of pharmacological activities such as antioxidant,^41^ anti‐inflammatory pain,^42^ hepatoprotective, antitumor, anti‐arthritic and acetylcholinesterase inhibitory effects. Additionally, 5,7‐dimethoxycoumarin and scopoletin have displayed potential binding affinity for the receptors used. To establish the efficacy of these two phytochemicals as potential drug candidates, the pharmacokinetics properties of these compounds were investigated through SwissADME. In this evaluation the drugs followed Lipinski's rule of five suggesting that they are potential medicinal constituents of *C. velutina* bark. Furthermore, to establish these phytochemicals as effective drug substances, PASS prediction was conducted using the PASS online tool which determines the probability of biological properties by comparing P_a_ (probability of activity) and P_i_ (probability of inactivity) of the tested substances. In PASS analysis, the probability of biological activities for scopoletin and 5,7‐dimethoxycoumarin conformed with the pharmacological actions found in literature reviews, including anti‐nociceptive, antipyretic, antioxidant and anti‐inflammatory actions, with scopoletin showing more efficacy than 5,7‐ dimethoxycoumarin.^4^

We have shown that the methanol extract of *C. velutina* bark possesses potential activity against pain, pyrexia and oxidation and a moderate effect on cytotoxicity. *C. velutina* is considered a highly potent traditional medicine and this was confirmed through our computer‐aided molecular docking, pharmacokinetics properties and pass prediction analysis.

## 5 CONCLUSIONS

The results of this study show that MECVB, evaluated through in vivo, in vitro and in silico assays, produces several pharmacological effects. The methanol extract of *C. velutina* bark demonstrated significant analgesic, antipyretic and anti‐oxidative properties and a moderate cytotoxic effect. In addition, phytochemicals present in the extract such as sitosterol, 5,7‐dimethoxycoumarin and scopoletin exhibited a promising binding affinity against selected receptors responsible for pain, fever and oxidation, and ADME/T and pass prediction analysis indicated that scopoletin and 5,7‐dimethoxycoumarin are potential drug candidates. Further more detailed investigations of this extract and the isolation and identification of the major bioactive compounds of MECVB are highly recommended for further study.

## AUTHOR CONTRIBUTIONS

Israt Jahan, Sanjida Sharmin and A. S. M. Ali Reza conceptualized and designed the research. Israt Jahan, Shahenur Alam Sakib and Sanjida Sharmin developed the methodology. Israt Jahan, Shahenur Alam Sakib, Najmul Alam and Mohuya Majumder carried out all the experiment, data collection and investigation. A. S. M. Ali Reza and Sanjida Sharmin supervised whole investigation. Israt Jahan drafted the manuscript. A. S. M. Ali Reza and Sanjida Sharmin revised and edited the final version of manuscript. All authors read and approved the final manuscript.

## ETHICS STATEMENT

Protocol used in this study for the use of mice as animal model for pharmacological research was approved by the Department of Pharmacy, International Islamic University Chittagong Ethical committee (Pharm/P&D‐158/14‐20).

## CONFLICT OF INTEREST

None to declare.
